# Supplementary material for: Single Cell Transcriptomic and Chromatin Profiles Suggest Layer Vb Is the Only Layer With Shared Excitatory Cell Types in the Medial and Lateral Entorhinal Cortex
Source: Front Neural Circuits. 2022 Jan 26;15:806154. doi: 10.3389/fncir.2021.806154 (PMC8826650; doi:10.3389/fncir.2021.806154)
Supplement: Supplementary file 14 [file Data_Sheet_1.ZIP › LEC_Sup_vs_MEC_Sup_Motifs/homerResults/motif12.similar.html]

motif12

## Information for motif12

G
A
C
T
C
T
G
A
A
G
T
C
C
G
T
A
T
C
A
G
G
C
A
T
C
G
T
A
T
C
G
A
T
C
G
A
T
A
G
C
G
T
C
A
C
T
G
A
  
Reverse Opposite:  

G
A
C
T
C
A
G
T
A
T
C
G
A
G
C
T
A
G
C
T
G
C
A
T
C
G
T
A
A
G
T
C
G
C
A
T
C
T
A
G
G
A
C
T
C
T
G
A
  

|  |  |
| --- | --- |
| p-value: | 1e-22 |
| log p-value: | -5.097e+01 |
| Information Content per bp: | 1.690 |
| Number of Target Sequences with motif | 1337.0 |
| Percentage of Target Sequences with motif | 10.03% |
| Number of Background Sequences with motif | 1529.6 |
| Percentage of Background Sequences with motif | 7.68% |
| Average Position of motif in Targets | 316.0 +/- 241.2bp |
| Average Position of motif in Background | 357.8 +/- 278.1bp |
| Strand Bias (log2 ratio + to - strand density) | -0.0 |
| Multiplicity (# of sites on avg that occur together) | 1.07 |
| Motif File: | file (matrix) reverse opposite |

### Similar de novo motifs found

|  |  |  |  |  |  |  |  |
| --- | --- | --- | --- | --- | --- | --- | --- |
| Rank | Match Score | Redundant Motif | P-value | log P-value | % of Targets | % of Background | Motif file |
| 1 | 0.620 | A G T C A C G T A C G T C G T A A G T C C G T A A C T G A C G T C G T A A C T G | 1e-5 | -11.805086 | 0.16% | 0.05% | motif file (matrix) |
